# How do deep-learning models generalize across populations? Cross-ethnicity generalization of COPD detection

**DOI:** 10.1186/s13244-024-01781-x

**Published:** 2024-08-07

**Authors:** Silvia D. Almeida, Tobias Norajitra, Carsten T. Lüth, Tassilo Wald, Vivienn Weru, Marco Nolden, Paul F. Jäger, Oyunbileg von Stackelberg, Claus Peter Heußel, Oliver Weinheimer, Jürgen Biederer, Hans-Ulrich Kauczor, Klaus Maier-Hein

**Affiliations:** 1 Division of Medical Image Computing, German Cancer Research Center (DKFZ), Heidelberg, Germany; 2 Translational Lung Research Center Heidelberg (TLRC), Member of the German Center for Lung Research (DZL), Heidelberg, Germany; 3 Medical Faculty, Heidelberg University, Heidelberg, Germany; 4 National Center for Tumor Diseases (NCT), NCT Heidelberg, a partnership between DKFZ and Heidelberg University Medical Center, Heidelberg, Germany; 5 Interactive Machine Learning Group (IML), German Cancer Research Center (DKFZ), Heidelberg, Germany; 6 Helmholtz Imaging, German Cancer Research Center (DKFZ), Heidelberg, Germany; 7 Division of Biostatistics, German Cancer Research Center (DKFZ), Heidelberg, Germany; 8 Pattern Analysis and Learning Group, Radiation Oncology, Heidelberg University Hospital, Heidelberg, Germany; 9 Diagnostic and Interventional Radiology, University Hospital, Heidelberg, Germany; 10 Diagnostic and Interventional Radiology with Nuclear Medicine, Thoraxklinik at University Hospital, Heidelberg, Germany; 11 University of Latvia, Faculty of Medicine, Raina Bulvaris 19, Riga, LV-1586 Latvia; 12 Christian-Albrechts-Universität zu Kiel, Faculty of Medicine, D-24098 Kiel, Germany

**Keywords:** Chronic obstructive pulmonary disease, Deep learning, Artificial intelligence, Computed tomography, Ethnicity

## Abstract

**Objectives:**

To evaluate the performance and potential biases of deep-learning models in detecting chronic obstructive pulmonary disease (COPD) on chest CT scans across different ethnic groups, specifically non-Hispanic White (NHW) and African American (AA) populations.

**Materials and methods:**

Inspiratory chest CT and clinical data from 7549 Genetic epidemiology of COPD individuals (mean age 62 years old, 56–69 interquartile range), including 5240 NHW and 2309 AA individuals, were retrospectively analyzed. Several factors influencing COPD binary classification performance on different ethnic populations were examined: (1) effects of training population: NHW-only, AA-only, balanced set (half NHW, half AA) and the entire set (NHW + AA all); (2) learning strategy: three supervised learning (SL) vs. three self-supervised learning (SSL) methods. Distribution shifts across ethnicity were further assessed for the top-performing methods.

**Results:**

The learning strategy significantly influenced model performance, with SSL methods achieving higher performances compared to SL methods (*p* < 0.001), across all training configurations. Training on balanced datasets containing NHW and AA individuals resulted in improved model performance compared to population-specific datasets. Distribution shifts were found between ethnicities for the same health status, particularly when models were trained on nearest-neighbor contrastive SSL. Training on a balanced dataset resulted in fewer distribution shifts across ethnicity and health status, highlighting its efficacy in reducing biases.

**Conclusion:**

Our findings demonstrate that utilizing SSL methods and training on large and balanced datasets can enhance COPD detection model performance and reduce biases across diverse ethnic populations. These findings emphasize the importance of equitable AI-driven healthcare solutions for COPD diagnosis.

**Critical relevance statement:**

Self-supervised learning coupled with balanced datasets significantly improves COPD detection model performance, addressing biases across diverse ethnic populations and emphasizing the crucial role of equitable AI-driven healthcare solutions.

**Key Points:**

- Self-supervised learning methods outperform supervised learning methods, showing higher AUC values (*p* < 0.001).
- Balanced datasets with non-Hispanic White and African American individuals improve model performance.
- Training on diverse datasets enhances COPD detection accuracy.
- Ethnically diverse datasets reduce bias in COPD detection models.
- SimCLR models mitigate biases in COPD detection across ethnicities.

**Graphical Abstract:**

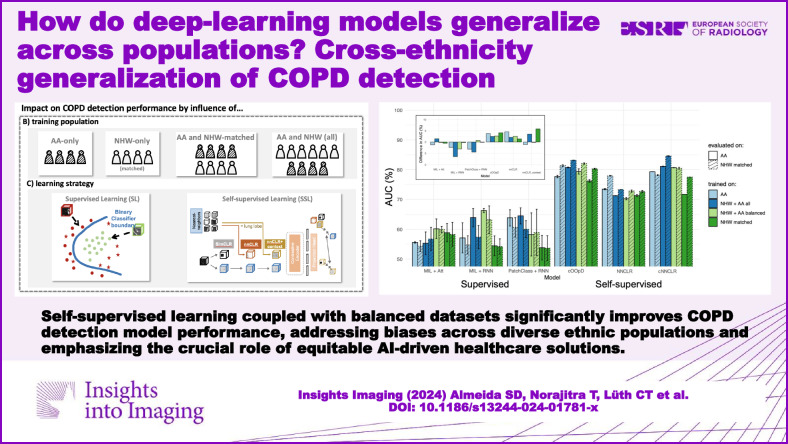

## Introduction

Chronic obstructive pulmonary disease (COPD) poses a significant challenge in healthcare settings due to its non-reversible airway and/or alveolar abnormalities, leading to persistent airflow obstruction. Despite its global prevalence of 10.3% [1], COPD remains underdiagnosed and misdiagnosed [2], necessitating improved diagnostic strategies. The complexity of COPD diagnosis arises from its diverse clinical presentations influenced by biological, socioeconomic, and cultural factors, with racial and ethnic disparities further complicating management.

Recent reports from 2021 in the US reveal COPD prevalence at 6.2% in African American (AA) and non-Hispanic Black individuals, slightly lower than 6.5% in non-Hispanic Whites (NHW) and notably higher than 3.9% in Latino individuals [3]. Cross-sectional studies consistently show AA individuals have lower lung function, up to 10–15% lower forced expiratory volume in 1 s (FEV1)) [4, 5], attributed in part to anthropometric factors [4, 6]. COPD disparities extend to health-related quality of life, dyspnea severity, exercise capacity, and exacerbation rates, with AA individuals experiencing worsened outcomes compared to NHW [7, 8]. Imaging findings reflect these differences, with AA individuals showing less severe emphysema on CT scans despite matched lung function impairments [9]. While race adjustments in spirometry reference equations have historically addressed these differences, recent perspectives advocate for race-neutral approaches to reduce potential biases in diagnosis and treatment, particularly in vulnerable populations [10–15]. This evolving perspective necessitates a reconsideration of established COPD diagnostic practices that may perpetuate racial or ethnic bias.

Amidst these challenges, the emergence of artificial intelligence has offered promising avenues for COPD diagnosis and management. Particularly on the imaging diagnosis front, deep learning (DL) has played a crucial role in COPD early diagnosis and improved outcomes [16–23]. However, concerns about potential racial bias in AI detection models have also surfaced as their capabilities unfold.

Recent studies [24, 25] suggest that rather than mitigating bias, these AI models might exacerbate and perpetuate unfairness, particularly against specific subpopulations. The mechanisms through which bias is perpetuated are multifaceted. During training, datasets may inadvertently underrepresent certain patient groups or contain harmful correlations, leading to a distortion of model outcomes. What amplifies the significance of these concerns is the realization that human biases are encapsulated in the target labels used to train these models [26]. Besides, the algorithm design may also have a higher tendency to learn and propagate such biases. Among the main categories of algorithm design are supervised learning (SL) and self-supervised learning (SSL) models. SL methods can inherit biases present in the labeled datasets [27], potentially perpetuating disparities in disease detection [25, 28, 29]. SSL, on the other hand, are less susceptible to biases inherent in labeled data, as they rely on learning representations directly from unlabeled data, often through pretext tasks. This independence from biased labels is a significant advantage, potentially reducing the risk of perpetuating biases present in annotated datasets. However, it’s crucial to note that SSL can still learn biases from the data itself, as well as from the design of the SSL task chosen. Even within the broader category of un-/self-supervised learning, state-of-the-art models may, to some extent, still harbor biases associated with learned associations from the data [26, 30].

Despite the growing significance of the issue, previous research has largely overlooked the potential ethnic biases encoded in common COPD imaging detection models, whether they employ SL or SSL techniques. Furthermore, the impact of such biases on the performance of these models remains unexplored.

In the face of this complex, multicausal issue, we investigated how COPD predictive models on chest CT, whether supervised or self-supervised, generalize across different ethnic populations. This exploration is specifically defined within the context of the largest COPD imaging dataset, Genetic epidemiology of COPD (COPDGene), serving as the focal point for our comprehensive inquiry.

Specifically, our exploration unfolds through three pivotal research questions:

- Research Question 1 (RQ1): To what extent do NHW and AA experience similar prediction performance when COPD detection models are trained on large-scale datasets?
- Research Question 2 (RQ2): What is the impact of the training population choice on the variations in test accuracies between NHW and AA? This involves assessing models trained exclusively on AA, NHW, and a balanced set comprising equal proportions of both.
- Research Question 3 (RQ3): If differences exist, are these smaller for SSL methods?

Examining the potential for unfairness in DL algorithms, whether due to the underrepresentation of minority populations in the training set or by the algorithm itself, is the first step for a comprehensive understanding of the intricate relationship between training population dynamics and algorithmic fairness in the realm of COPD predictive models.

## Materials and methods

### Study sample

Our study retrospectively analyzed COPDGene phase 1 study [31] (clinicaltrials.gov, NCT00608764; http://www.copdgene.org/), which recruited current and former self-reported NHW and AA smokers (≥ 10 pack-years), aged 45–80 years, between 2008 and 2011. Paired chest CT in inspiration (Insp) and expiration (Exp), pulmonary function tests, and questionnaires were collected per subject. Imaging data was acquired from different scanners and different manufacturers. Specific image acquisitions vary on the scanner model, which is available in [31, 32].

To streamline the analysis and maintain simplicity, only inspiratory images were included in this study, as contrastive tasks have demonstrated robustness even without the inclusion of expiratory images [20]. Pre-processing strategies followed the description of [20, 21].

### Subpopulation matching and data split

Differences in COPD prediction between NHW and AA, if any, could be related to confounding effects of demographic and risk factors variables. To limit the influence of such factors, a population of NHW was selected to match the AA population (NHW-matched), based on individuals with the same age, gender, and smoking duration (years). Having this in mind, to explore the effects of the training population, COPD prediction models were trained on the entire dataset (NHW and AA), AA only, NHW-matched only, and on a perfectly balanced set (half NHW-matched + half AA).

Differences in COPD prediction were evaluated on the test set splits of AA only and NHW-matched only.

Data splits for training, validation, and testing followed the same strategy as in [20, 21], now applying it to the AA set.

### COPD model prediction

Aiming to investigate the impact of SL and SSL on COPD binary classification performance, several models were evaluated.

#### Supervised learning (SL) models

For the evaluation of SL methods, we adopted three well-established voxel-based approaches: end-to-end patch classifier with a recurrent neural network (PatClass + RNN); multiple instance learning (MIL) with RNN as aggregation (MIL + RNN); attention-based MIL (MIL + Att). All methods are thoroughly described in the Supplementary Materials S-1.

#### Self-supervised learning (SSL) models

For the evaluation of SSL methods, three self-supervised contrastive tasks were compared (SimCLR, NNCLR, and context-aware NNCLR), having a fixed anomaly detection approach as a downstream task. These models are based on a recently proposed self-supervised anomaly detection method by Almeida SD et al [20, 21] (cOOpD). This approach is founded on modeling the distribution of normal-lung regions utilizing contrastive latent representations and identifying deviations from this distribution as COPD-anomalous samples. In their approach, SimCLR [33] was used as the self-supervised contrastive model, as a pretext task to extract highly informative latent features per lung region. Subsequently, a generative model was applied to healthy regions from normal-lung-function subjects to discern the distribution of “normality.” Out-of-distribution samples were assigned an anomaly score based on the negative log likelihood, enabling the identification of COPD regions. Patient-level labels were obtained by aggregating local-level scores.

To further enhance the richness of latent representations and extend beyond single instance positives, we adapted and compared the Almeida SD et al cOOpD method with two self-supervised pretext methods: nearest-neighbor contrastive learning approach (NNCLR) [34] and to a novel Context-Aware NNCLR (cNNCLR).

The NNCLR method introduces diversity in positive pairs by incorporating nearest neighbors sampled from a memory bank, aiming to increase the richness of latent representations and overcome limitations of pre-defined data augmentations.

The novel cNNCLR adaptation addresses concerns regarding disease-related sample selection by enforcing that nearest neighbors come from the same lung lobe and patient, leveraging spatial information for refined representations. This adaptation is particularly important given the subtle and heterogeneous pathological patterns observed in COPD.

For both NNCLR and cNNCLR, implementation configurations followed established strategies for random augmentations, encoder selection, and memory bank size, ensuring consistency with previous work [34]. The same downstream task as the original Almeida SD et al [20, 21] method was employed for all self-supervised pretext tasks. Further details about the method and implementations are available in the Supplementary Materials S-2 and S-3. Supplementary Fig. 1 illustrates the main differences between NNCLR and cNNCLR.

The code for the self-supervised models is available on a public repository on GitHub (https://github.com/MIC-DKFZ/cOOpD).

### Statistical analysis

Model performance was assessed using the Area Under the Receiver Operator Curve (AUC) as the main evaluation metric. The Area Under the Precision Recall Curve (AUPRC) is also reported. Further details are available in Supplementary Materials S-4. Differences in test performance between AA and NHW were measured based on the AUROC.

Multiple linear regression analysis was performed to predict the AUC, based on the following independent variables: type of learning method (SL vs SSL), training configuration (AA, NHW, AA + NHW, AA + NHW balanced), and evaluation population (AA-only and NHW-only). Multiple linear regression was chosen to quantify the contribution of each predictor and their interactions, providing a comprehensive analysis of the effects of the learning method, training configuration, and evaluation population on the AUC. Corrections for multiple comparisons were addressed using the Holm-Bonferroni method.

The distribution of the anomaly scores generated by the SSL methods was compared using the Kolmogorov–Smirnov Test. The hypothesis is that the distributions of the individual binary classes (diseased/healthy) should be identical, independently of the ethnicity. Benjamini–Yekutieli correction was applied to the *p* values.

Statistical analyses were performed with R (version 4.2.3; R Foundation for Statistical Computing). A *p* value of < 0.05 was considered statistically significant.

## Results

### Dataset characteristics

Table 1 presents the demographic data and lung function parameters of the study sample employed in this study, divided by ethnicity (AA and NHW). An extra column is provided for the NHW population matched to AA (NHW-matched). Patient characteristics are then divided by training, evaluation, and test sets. Overall, this study comprised 7549 COPDGene individuals (mean age 62 years old, 56–69 interquartile range), from which 5240 were NHW and 2309 were AA.

**Table 1:** Demographic data and functional parameters for the analyzed COPDGene study sample, divided by ethnicity and by dataset split (training, evaluation, and testing)

Attribute	Non-Hispanic White (NHW)	African American (AA)	NHW-matched to AA
All data			
*N* Patients	5240	2309	2312
M (*N*)	2841	1297	1242
F (*N*)	2399	1012	1070
Age (y) (mean (IQR))	62 (56–69)	55 (49–59)	58 (51–63)
BMI (mean (SD))	28.4 (5.8)	28.6 (6.3)	28.6 (5.8)
Smoking habits			
Never-smoker (*N* (%))	105 (2.0%)	7 (0.3%)	0 (0%)
Former smoker (*N* (%))	3158 (60.3%)	485 (21.0%)	1072 (46.4%)
Current smokers (*N* (%))	1977 (37.7%)	1817 (78.7%)	1240 (53.6%)
Smoking duration (y) (mean (SD))	36 (12)	36 (9)	37 (9)
Spirometry			
FEV1%_pred (mean (SD))	74.9 (26.9)	83.9 (25.3)	82.3 (24.6)
FEV1/FVC (mean (SD))	0.6 (0.2)	0.7 (0.2)	0.7 (0.2)
Imaging			
LAA-950% (mean (SD))	7.9 (10.5)	4.5 (8.3)	5.4 (6.7)
LAA-878% (mean (SD))	25.6 (20.5)	18.8 (18.9)	19.1 (18.2)
Training data			
*N* Patients	3144	1384	1386
M (*N*)	1699	776	741
F (*N*)	1445	608	645
Age (y) (mean (IQR))	63 (55–69)	55 (49–59)	57 (51–63)
BMI (mean (SD))	28.3 (5.7)	28.6 (6.3)	28.6 (5.8)
Smoking habits			
Never-smoker (*N* (%))	63 (2.0%)	3 (0.2%)	0 (0.0%)
Former smoker (*N* (%))	1862 (59.2%)	303 (21.9%)	615 (44.4%)
Current smokers (*N* (%))	1219 (38.8%)	1078 (77.9%)	771 (55.6%)
Smoking duration (y) (mean (SD))	36 (12)	36 (9)	37 (9)
Spirometry			
FEV1%_pred (mean (SD))	75.4 (26.8)	83.9 (25.1)	82.2 (24.7)
FEV1/FVC (mean (SD))	0.6 (0.2)	0.7 (0.2)	0.7 (0.2)
Imaging			
LAA-950% (mean (SD))	7.8 (10.4)	4.5 (8.6)	5.5 (9.0)
LAA-878% (mean (SD))	25.4 (20.4)	18.7 (18.9)	19.3 (18.5)
Validation data			
*N* Patients	786	347	347
M (*N*)	450	198	199
F (*N*)	336	149	148
Age (y) (mean (IQR))	63 (56–69)	55 (49–59)	58 (52–63)
BMI (mean (SD))	28.3 (5.7)	28.5 (6.2)	28.6 (5.6)
Smoking habits			
Never-smoker (*N* (%))	13 (1.7%)	0 (0.0%)	0 (0.0%)
Former smoker (*N* (%))	479 (60.9%)	73 (21.0%)	165 (47.6%)
Current smokers (*N* (%))	294 (37.4%)	274 (79.0%)	182 (52.4%)
Smoking duration (y) (mean (SD))	36 (12)	37 (9)	37 (9)
Spirometry			
FEV1%_pred (mean (SD))	74.2 (26.8)	82.5 (25.2)	82.1 (25.2)
FEV1/FVC (mean (SD))	0.6 (0.2)	0.7 (0.1)	0.7 (0.2)
Imaging			
LAA-950% (mean (SD))	8.0 (10.6)	4.9 (8.8)	4.9 (8.0)
LAA-878% (mean (SD))	25.7 (20.3)	20.0 (20.3)	19.0 (17.6)
Test data			
*N* Patients	1310	578	579
M (*N*)	692	323	302
F (*N*)	618	255	277
Age (y) (mean (IQR))	63 (56–69)	55 (49–59)	58 (52–63)
BMI (mean (SD))	28.1 (5.7)	29.0 (6.5)	28.4 (6.2)
Smoking habits			
Never-smoker (*N* (%))	29 (2.2%)	4 (0.7%)	0 (0.0%)
Former smoker (*N* (%))	817 (62.4%)	109 (18.9%)	292 (50.4%)
Current smokers (*N* (%))	464 (35.4%)	465 (80.4%)	287 (49.6%)
Smoking duration (y) (mean (SD))	36 (12)	36 (9)	37 (9)
Spirometry			
FEV1%_pred (mean (SD))	74.2 (27.1)	84.7 (25.8)	82.8 (24.2)
FEV1/FVC (mean (SD))	0.6 (0.2)	0.7 (0.1)	0.7 (0.2)
Imaging			
LAA-950% (mean (SD))	8.1 (10.6)	4.1 (7.5)	5.3 (8.2)
LAA-878% (mean (SD))	26.1 (21.1)	18.2 (18.0)	18.8 (18.0)

Attenuation percentages were measured by VIDA Diagnostics

*COPDGene* genetic epidemiology of COPD, *N* number, *sd* standard deviation, *y* years, *BMI* body mass index, *FEV*_*1*_ forced expiratory volume in 1 s, *FEV*_*1*_*/FVC* FEV_1_-to-forced vital capacity ratio, *LAA-950%* percentage of LAA under −950 HU, *LAA-856%* percentage of LAA under −856 HU

### Model performance

**Fig. 1:**
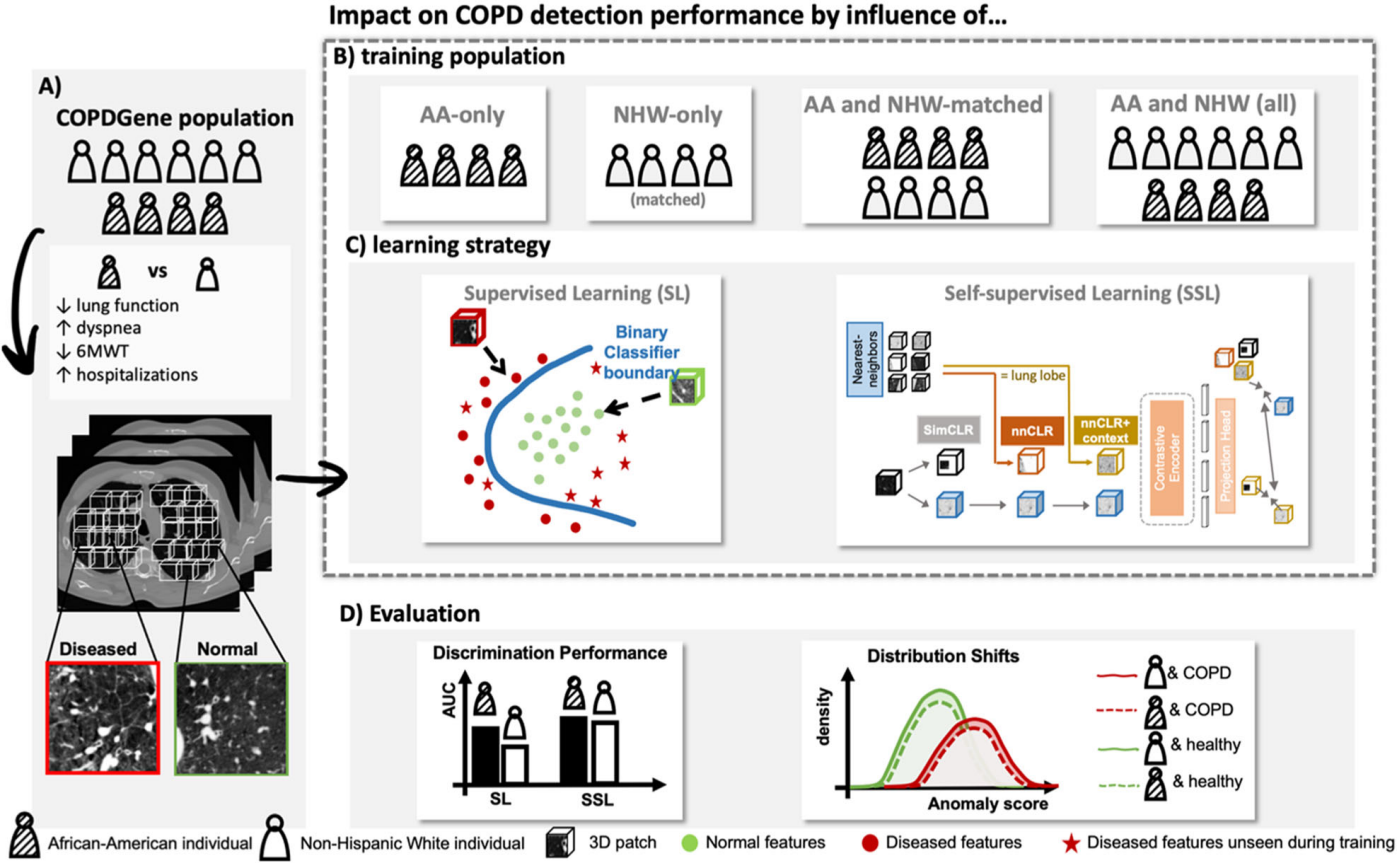
The schematic workflow of this study. **A** Main differences in COPD-related clinical characteristics between non-Hispanic Whites (NHW) and African-Americans (AA) and visual representation of normal and diseased regions on chest CT. The impact on COPD detection performance was assessed by the influence of two factors: **B** Training population (AA-only, NHW-matched-only, AA and NHW-matched, and AA and NHW all) and (**C**) Learning strategy (supervised learning [SL] and self-supervised learning [SSL]). **D** The impact is evaluated by comparing the Area Under the Receiver Operator Curve (AUC) per training configuration and learning strategy and by assessing the differences in distributions produced by the top-performing method

The differences in performance in terms of the AUC across models, training, and evaluation patient subgroups are summarized in Fig. 1 and in Supplementary Table 1. SSL methods generally outperform SL methods, with SL methods showing a lower average performance, irrespective of the training and evaluation configuration. Furthermore, AUC shows higher dispersion in SL models than in SSL. Overall, the best-performing combination is the NNCLR with the context framework applied to the large-scale dataset (NHW + AA all), followed by SimCLR.

Table 2 presents results from the multiple linear regression model. Interactions between the various predictors were also tested but since they were not significant, the model was refitted without interactions. As indicated in Table 2, the F-statistic *p* value is significant implying that at least one of the predictors (the type of learning, training configuration, and evaluation population) is significantly associated with the AUC. The overall coefficient of determination (*R*^2^) indicates how much the model explains the variance of the AUC. The contribution of each predictor (type of learning, training configuration, and evaluation population) on the dependent variable (AUC) is indicated by the respective β values and *p* values.

**Table 2:** Multiple linear regression analysis to predict the main performance metric (AUC) with the following as independent variables: type of learning method (supervised vs self-supervised), training configuration (AA only, NHW-matched only, AA + NHW all, AA + NHW balanced) and evaluation population (NHW-matched only and AA only)

Multiple linear regression analysis		
*F*(5, 42) = 61.18, *p* < 2e-16, *R*^2^ = 0.88, adj *R*^2^ = 0.86		
Independent variable	β	*p*
Type of Learning (supervised vs self-supervised)	−18.90	< 2e-16 *
Training configuration (AA vs NHW + AA all)	−1.49	0.34
Training configuration (NHW + AA balanced vs NHW + AA all)	0.03	0.98
Training configuration (NHW matched vs NHW + AA all)	−4.09	0.01*
Evaluation population (NHW-matched vs AA)	0.48	0.67

The second line provides information on the model fit, including the F-statistic. Reference categories are underlined

* *p* value was significant after Holm-Bonferroni correction for multiple comparisons

SL methods had a significantly lower AUC (β = −18.90, *p* < 2e-16) compared to SSL, holding the training configuration and evaluation population constant. Training on the NHW-matched population resulted in a statistically significant lower AUC than training on NHW + AA all population (β = −4.09, *p* = 0.01). Although not significant, training on the AA-only population showed a lower AUC trend than the reference NHW + AA all population. No differences were found for training on the balanced set (half NHW-matched + half AA) compared to training on the entire population (NHW + AA all) holding the type of learning and evaluation population constant. Similarly, no differences were found between the evaluation populations, holding the type of learning and the training configuration constant.

### RQ1: To what extent do NHW and AA experience similar prediction performance when COPD detection models are trained on large-scale datasets?

No statistically significant difference was found between the evaluation populations when holding the other predictors constant. This indicates that NHW and AA individuals experience similar prediction performance, independently of the learning strategy and training configuration. Still, SL models trained with diverse data sources (NHW + AA all) exhibited larger mean performance differences between NHW and AA populations. Furthermore, this same training configuration (NHW + AA all) exhibited higher AUC than population-specific configurations (NHW-matched *p* = 0.01, tendency for AA-only n.s.), while no difference was found when compared with the balanced set (half NHW-matched + half AA). Therefore, although no difference was found for the COPD detection performance between AA and NHW, the performance is higher when models are trained on the entire (NHW + AA all) or on a balanced set (half NHW-matched + half AA).

### RQ2: What impact does the choice of the training population have on the differences in test accuracies between NHW and AA?

Regardless of the training population, SL consistently demonstrates higher AUC when evaluated on the AA population, compared to NHW individuals. For SSL, there are instances where the AUC mean is higher when training on a population matched with the evaluation population (e.g., NHW-matched when evaluating on NHW). This effect is consistent across all models and configurations, except for NNCLR models. Although no statistically significant difference was found for the evaluation population, the training configuration has an impact on the overall AUC: including both NHW and AA patients in the training set improves the model’s performance on both populations compared to training on a population-specific dataset.

### RQ3: If differences exist, are these smaller for self-supervised methods?

**Fig. 2:**
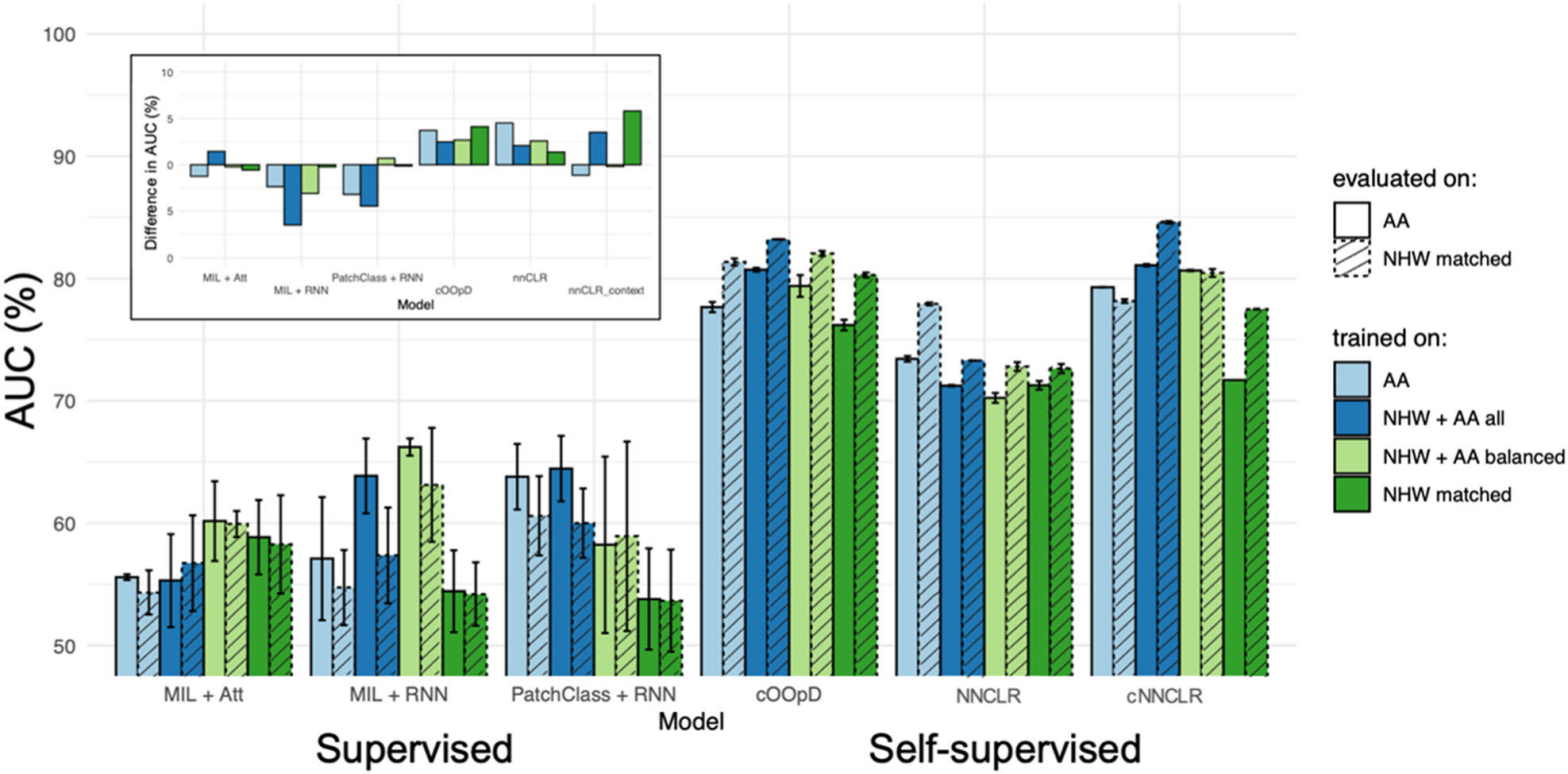
Supervised models show lower performance and higher uncertainty compared to self-supervised models. Comparison of COPD prediction performance across supervised (MIL + Att, MIL + RNN, PatchClass + RNN) and self-supervised (cOOpD, NNCLR, cNNCLR) models and across training and evaluation sub-ethnicity groups. Training subgroups are represented by color, while evaluation subgroups by linetype. Average classification performance across ethnic subgroups is shown in terms of the AUC (%), with error bars representing min–max values. The barplot on the top left corner represents mean AUC differences (NHW - AA) between models. Thus, positive bars represent higher prediction performance for models evaluated on NHW, compared to models evaluated on AA

Figure 2 illustrates that SL generally exhibits lower performance and higher uncertainty in COPD prediction compared to SSL. Furthermore, SL trained on the entire population tends to demonstrate higher pronounced differences in performance between NHW and AA individuals. Conversely, SSL, while achieving higher mean AUC overall (*p* < 2e-16), also reveals greater discrepancies between ethnicities, particularly when trained on other population configurations.

### Do the distributions of anomaly scores generated by SSL exhibit bias, and is there evidence to support the hypothesis that the distributions of individual binary classes (diseased/healthy) are identical, irrespective of ethnicity?

**Fig. 3:**
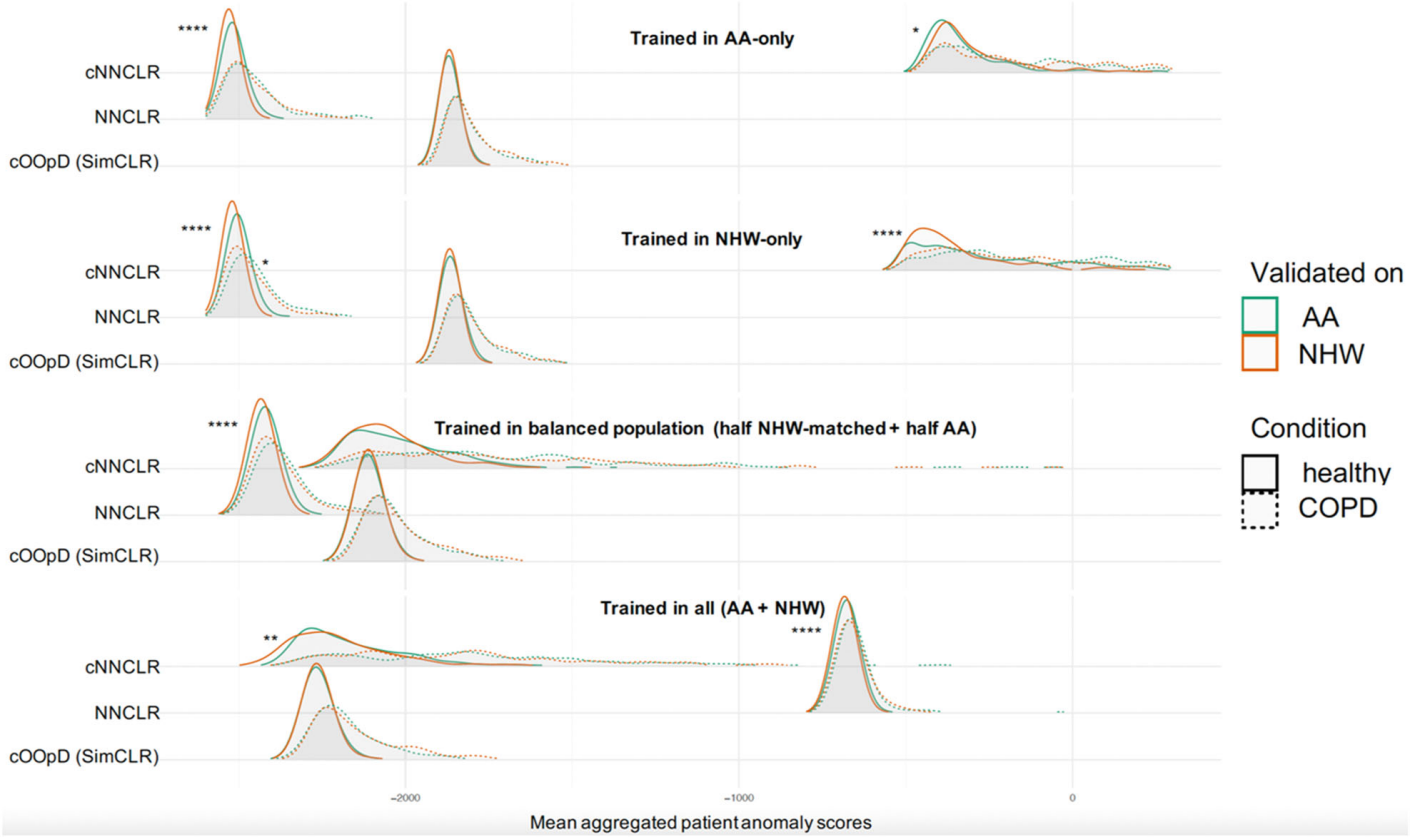
Training on a matched, balanced population (half NHW-matched + half AA) shows fewer distribution shifts across ethnicities, for the same health condition. The SSL cOOpD model is revealed to be the best generalizable. Distribution shifts in patient-wise anomaly scores. Distributions of healthy (green) and COPD cases (orange) for AA individuals (full line) and NHW individuals (dotted line) are plotted across self-supervised models (cOOpD, nnCLR, cNNCLR), for four training configurations (AA-only, NHW-only, half NHW-matched + half AA, AA + NHW all). The plots were generated using all individuals in the test set group. Statistically significant differences, noted by “*” (**** < 0.0001, *** < 0.001, ** < 0.01, * < 0.05), measured by the Kolmogorov–Smirnov Test are displayed per condition: healthy (left) and for COPD (right)

Figure 3 displays the differences between ethnicities (AA vs. NHW) across anomaly score distributions of healthy and diseased subjects, for different training configurations and SSL models. Particularly, NNCLR with context-aware training (cNNCLR) exhibited more prominent and larger differences, with clear shifts between AA and NHW patients observed in the subgroup distributions for COPD/healthy. Conversely, no obvious separation was observed for cOOpD across any of the training configurations. Notably, training on the entire dataset (NHW + AA all) resulted in minimal visually relevant differences.

As presented in Table 3, the statistical analysis confirmed these qualitative observations. No statistically significant differences were found for cOOpD models, indicating similar distributions for AA and NHW, both for healthy and diseased patients, across different training configurations. For NNCLR, on the other hand, significant differences were found between the marginal distributions for AA and NHW healthy patients across all training configurations (*p* < 0.0001) for all. For diseased patients, no evidence of differences was found, except when training in NHW-only (*p* = 0.03). Finally, for cNNCLR, differences in distributions were found between ethnicities of healthy individuals when models were trained in AA-only (*p* = 0.02), NHW-only (*p* < 0.0001), and on the entire dataset (*p* = 0.003). No differences were found in cNNCLR for the diseased patient-wise anomaly score distribution in all cases and for healthy individuals when the model was trained on the balanced set (half NHW-matched + half AA).

**Table 3:** Kolmogorov–Smirnov Tests for Comparing Distributions of ethnic evaluation populations across patient-wise anomaly score distributions for self-supervised models (cOOpD, NNCLR, cNNCLR)

Self-supervised model	Trained on							
	AA-only		NHW-only (matched)		half NHW-matched + half AA (balanced)		AA + NHW (all)	
	Healthy	COPD	Healthy	COPD	Healthy	COPD	Healthy	COPD
cOOpD	> 0.99	> 0.99	> 0.99	> 0.99	> 0.99	> 0.99	> 0.99	> 0.99
NNCLR	< 0.0001****	> 0.99	< 0.0001****	0.03*	< 0.0001****	0.19	< 0.0001****	0.09
cNNCLR	0.02*	> 0.99	< 0.0001****	0.11	0.05	> 0.99	0.003**	> 0.99

**** < 0.0001, ** < 0.01, * < 0.05

## Discussion

In this study, we compared DL models for COPD detection on chest CT scans across ethnic groups. SSL outperformed SL methods (*p* < 0.001), yielding higher AUC and lower uncertainty. Training on the entire COPDGene dataset produced better performance, with no significant differences compared to a balanced population. SL performed better on AA individuals, while SSL showed varying NHW-AA performance differences. However, SL trained on the full dataset exhibited larger performance gaps between AA and NHW. Including NHW and AA-matched patients improved performance and reduced differences, favoring SSL methods. In addition, SSL trained on balanced datasets showed more consistent anomaly score distributions across ethnicities, suggesting their potential to mitigate bias. These findings underscore the importance of considering ethnicity in model development and training to ensure equitable performance across diverse populations in COPD diagnosis.

While our study contributes significantly to understanding the performance and biases of DL models in COPD detection, it also sheds light on an important gap in the existing literature. The vast majority of fairness studies conducted to date have focused on pathology classification tasks within medical imaging [25, 35–40], with no attention paid to COPD diagnosis in minority classes. Despite the prevalence and significant healthcare burden associated with COPD, its diagnostic prediction performance across ethnicities remains understudied. Therefore, our work cannot be directly compared to other studies. However, studies from Glocker et al [25] and Seyyed-Kalantari et al [36] have evaluated bias in AI algorithms for various pathologies in chest X-rays. Parallelly to our findings, both studies highlight the presence of performance disparities and biases in AI models utilized for disease detection across various demographic subgroups, including biological sex, race, and, for the latter, socioeconomic status. Still, the effect of the training population and different types of learning strategies on pathology diagnosis has not been addressed.

Our findings also resonate with recent guidance from the American Thoracic Society (ATS) [41], which advocates for the adoption of race-neutral average reference equations in pulmonary function testing interpretation, while discouraging race and ethnicity adjustments. Our observations are consistent with these overarching goals, as, models trained on ethnic-specific datasets, exhibited, on average, larger differences in COPD prediction performance. On the other hand, on average, SSL exhibited fewer disparities in COPD prediction between different ethnic populations when models were trained on the entire or on balanced dataset. Our analysis of anomaly score distributions also revealed less statistically significant differences between ethnicities across healthy and diseased subjects when models are trained on the balanced dataset. This underscores the importance of leveraging ethnically diverse training datasets to enhance model robustness and mitigate potential biases.

The implications of our study are multifaceted and can inform future research and clinical practice in several key areas. First, our findings underscore the importance of evaluating DL models for medical applications across diverse demographic groups to ensure equitable performance and minimize biases. This highlights the need for comprehensive data collection efforts that include diverse populations to train models effectively and promote generalizability. Second, our study emphasizes the potential of SSL methods to mitigate biases and improve model performance in COPD detection. One possible reason for this improvement, over SL, is that SSL methods are likely circumventing biases that may be inherent in labeled datasets, thereby improving model generalization and reducing disparities across different demographic groups. SSL models excel in capturing nuanced patterns and variations in lung characteristics, including those influenced by demographic factors, leading to more robust and adaptable performance. Moreover, SSL mitigates the risk of overfitting to specific labeled examples, making it more resilient in real-world applications. In general, SSL can reduce the dependency on labor-intensive manual labeling and leverage the abundant unlabeled CT scans in the medical datasets, offering scalable solutions for improving COPD diagnosis and equity in healthcare outcomes. This suggests that investing in the development and evaluation of SSL approaches could yield significant benefits for improving COPD diagnostic accuracy and reducing disparities. In addition, our analysis underscores the importance of considering the choice of training data and its impact on model performance and bias. Finally, our study raises a critical consideration regarding the optimal balance between model performance and equity in healthcare outcomes. The choice between a lower-performing model with reduced disparities between ethnic groups or a higher-performing model with some differences between them warrants further examination in the context of improving equitable access to healthcare for diverse populations.

There are some limitations to our study worth reporting. While we rigorously matched the subgroups for comparison, it’s important to acknowledge the limitation regarding the inability to match other factors, such as the study site. Specifically, there were disproportionately fewer NHW individuals at study sites primarily serving AA individuals. Furthermore, while we focused on ethnicity as a key demographic variable, other factors such as socioeconomic status, education level, and environmental exposures were not addressed in our analysis. In addition, despite matching on smoking duration, discrepancies in smoking status (i.e., proportions of never-smokers, former smokers, and current smokers) between NHW and AA populations remain, influenced by differences in smoking initiation, cessation rates, cultural norms, and potential sampling variability within our study cohort. Future studies should aim to incorporate a more comprehensive set of demographic and clinical variables to better understand the complex interplay between patient characteristics and model performance.

In conclusion, our study highlights the significance of considering ethnicity in developing equitable COPD diagnostic models. We advocate for comprehensive data collection efforts and the exploration of SSL methods to mitigate biases and improve diagnostic accuracy across diverse populations, paving the way to ensuring equitable benefits for all population segments.

### Supplementary information

ELECTRONIC SUPPLEMENTARY MATERIAL

## Data Availability

Data generated by the authors or analyzed during the study are available at ClinicalTrials.gov Identifier: NCT00608764.

## Abbreviations

AA: African Americans
AUC: Area under the receiver operator curve
cNNCLR: Context-aware NNCLR
COPD: Chronic obstructive pulmonary disease
COPDGene: Genetic epidemiology of COPD
DL: Deep learning
NHW: Non-Hispanic Whites
SL: Supervised learning
SSL: Self-supervised learning
