# Supplementary material for: How do deep-learning models generalize across populations? Cross-ethnicity generalization of COPD detection
Source: Insights Imaging. 2024 Aug 7;15:198. doi: 10.1186/s13244-024-01781-x (PMC11306482; doi:10.1186/s13244-024-01781-x)
Supplement: Supplementary file 1 — ELECTRONIC SUPPLEMENTARY MATERIAL [file 13244_2024_1781_MOESM1_ESM.pdf]

How do deep-learning models generalize across populations? Cross-ethnicity generalization of COPD detection

ELECTRONIC SUPPLEMENTARY MATERIAL

All experiments were implemented in PyTorch and Pytorch-Lightning, using a NVIDIA GeForce RTX 2080 Ti GPU.

S-1. Compared supervised methods

For the evaluation of SL methods, we adopted three well-established voxel-based approaches: End-to-end Patch Classifier with a recurrent neural network (PatClass + RNN); Multiple Instance Learning (MIL) with RNN as aggregation (MIL + RNN); Attention-based MIL (MIL + Att).

S-1.1 PatClass+RNN

A supervised end-to-end Patch Classifier with a recurrent neural network (RNN) as aggregation to obtain patient-level scores. The model receives as input a 3D patch, having the same label as the global patient label (0 or 1). As output, the model produces a probability at the patch-level, of being diseased or not. This probability is then aggregated per patient. Several aggregation strategies were experimented, but RNN, as described by[1], showed to be the best performing one on the validation set. Here, the S=10% most suspicious patches were sequentially passed to the RNN to predict the final patient-level classification. As an encoder we used a 3D ResNet34, which was trained for 100 epochs, with a batch size of 64, using the Adam Optimizer, learning rate of 1e-4, Cosine Annealing[2], with cross entropy loss. As transformations, elastic, rotation, scaling, random cropping, mirroring, gaussian noise and gaussian blur were employed with a probability of 5%.

S-1.2 MIL+RNN

A supervised multiple instance learning (MIL) strategy with a recurrent neural network as aggregation, as proposed by[1]. Given a bag of instances (where the bag is a patient and the instances are 3D patches), all instances are classified and ranked according to their probability of being positive (diseased). If the bag is positive (which means that the patient- level label is diseased), the probability of being positive of the highest-ranked instance should be very close to 1; if the bag is negative (normal), the highest ranked instance should have a very low probability, approaching 0. Therefore, the task here is to learn the optimal instance (patch) level representation that can linearly discriminate patches that contribute to a diseased global label, from those patches that do not. The implementation details are as described in the original paper, with the exception of the encoder (3D ResNet34). S (probability) was set to 10%.

S-1.3 MIL+Att

A supervised MIL with an attention layer. Similar to the MIL+RNN method, this MIL builds on the same strategy but employs a latter layer with an attention mechanism. This layer provides a measure of the contribution of each patch to the overall patient label. The overall idea is identical to Sun et al. COPD detection model[3], except we build on the 3D patch notion, while they selected slices from the original CT. Overall configuration details were as described by[4], with the adaptations to 3D, and using a 3D ResNet34 as encoder.

S-2. Nearest-Neighbor Contrastive Learning Approaches

As SimCLR solely relies on transformations introduced by pre-defined data augmentations on the same sample, it cannot link multiple samples potentially belonging to the same semantic class, which in turn might decrease its capacity to be invariant to large intra-class variations. In this approach, each patch is augmented twice with random transformations (positive pair). Loss is calculated under the assumption that the latent space of these random transformations is closer than other negative patches. However, these random transformations are limited and may not characterize the disease itself.

S-2.1. NNCLR: Nearest-Neighbor Contrastive Learning

Having this, we hypothesize that the improvement in performance can be achieved if diversity is introduced in the positive pairs, something that is not covered by pre-defined data augmentation schemes. Having this, nearest-neighbors could be useful to obtain more diverse positive pairs, in order to increase the richness of our latent representation and go beyond single instance positives. This requires keeping a support set of embeddings which is representative of the full data distribution.

This framework is called nearest-neighbor contrastive learning (NNCLR). Each patch is augmented twice with random transformations. One of the views is replaced with its nearest-neighbor (NN) in the latent space, sampled from a memory bank that is updated with every batch. Loss is calculated under the assumption that the memory bank represents the entire diversity of the training data and that a NN with some diversity can be found there - no longer limited to the set of transformations.

S-2.2 cNNCLR: context-aware Nearest-Neighbor Contrastive Learning

NNCLR may choose NN to discriminate that are not necessarily disease-related. For instance, centrilobular emphysema, the most common form of smoking-related emphysema, is typically upper lobe predominant. It begins in the central portion of the secondary pulmonary lobules, initially appearing as small holes that become more confluent as the disease progresses[41]. Pathological patterns are often subtle and heterogeneous, and anatomy-related. These abnormal changes are sensitive to specific lobe regions. Therefore, finding the nearest neighbor in the latent space does not necessarily mean the patches are spatially or disease connected.

For this reason, we have introduced context-aware NNCLR (cNNCLR). This is an adaptation of the NNCLR approach, where we force the nearest neighbor to come from the same lung lobe and same patient, if it exists in the memory bank. In early iterations where a certain lobe may be lacking, cNNCLR identifies the nearest neighbor without regard to location, progressively refining its representations as spatial information becomes available.

S-3. Implementation configurations for NNCLR and cNNCLR

We followed the same strategy of random augmentations described before[21], based on [42]: Non-linear transformation based on the Bezier curve, local-pixel shuffling and in- and out-painting. A maximum of 100 patches per patient was set for training the self-supervised contrastive tasks. As an encoder, the 3D ResNet-34 configuration was used. The memory bank size was set to 4096, as described in their original work[33]. The loss function employed was the InfoNCE (Info Noise Contrastive estimation), which uses categorical cross-entropy loss to identify the positive sample amongst a set of unrelated noise samples.

S-4. Model performance

Model performance for COPD binary classification was assessed using Area Under Receiver Operator Curve (AUC) and Area Under Precision Recall Curve (AUPRC) as the default multi-threshold metric for classification. AUC is used as the main evaluation metric since it is less sensitive to class balance changes. Final method configurations were tuned on the evaluation set, based on the highest AUC on three experiment runs. Supplementary Table 1 further shows all methods' performance on the internal and external test sets, for all inputs.

Supplementary table 1: Mean ± standard deviation of the Area Under the Receiver Operating Curve (AUC) and Area Under the Precision Recall Curve (AUPRC), as main performance metrics for COPD binary classification. Values are obtained per model (supervised learning [SL]: MIL + Att, MIL + RNN, PatchClass + RNN; self-supervised learning [SSL]: cOOpD, NNCLR, cNNCLR) and per training configuration (AA-only, NHWmatched-only, AA+NHW matched, AA+ NHW all), based on three test set runs on AA and NHW test set population.

Type	Model	Config	Trained on	Evaluated on	AUC (mean ± sd)	AUPRC (mean ± sd)
SL	MIL + Att	insp 0%	NHW matched	NHW matched	0.58 ± 0.04	0.59 ± 0.05
SL	MIL + Att	insp 0%	NHW matched	AA	0.61 ± 0.03	0.57 ± 0.05
SL	MIL + Att	insp 0%	AA	NHW matched	0.54 ± 0.02	0.50 ± 0.01
SL	MIL + Att	insp 0%	AA	AA	0.56 ± 0.00	0.52 ± 0.01
SL	MIL + Att	insp 0%	NHW + AA balanced	NHW matched	0.61 ± 0.01	0.58 ± 0.01
SL	MIL + Att	insp 0%	NHW + AA balanced	AA	0.62 ± 0.03	0.59 ± 0.02
SL	MIL + Att	insp 0%	NHW + AA all	NHW matched	0.56 ± 0.04	0.56 ± 0.02
SL	MIL + Att	insp 0%	NHW + AA all	AA	0.57 ± 0.04	0.53 ± 0.03
SL	MIL + RNN	insp 0%	NHW matched	NHW matched	0.54 ± 0.03	0.56 ± 0.08
SL	MIL + RNN	insp 0%	NHW matched	AA	0.53 ± 0.03	0.63 ± 0.04
SL	MIL + RNN	insp 0%	AA	NHW matched	0.56 ± 0.03	0.51 ± 0.04
SL	MIL + RNN	insp 0%	AA	AA	0.57 ± 0.05	0.54 ± 0.05
SL	MIL + RNN	insp 0%	NHW + AA balanced	NHW matched	0.64 ± 0.05	0.61 ± 0.03
SL	MIL + RNN	insp 0%	NHW + AA balanced	AA	0.66 ± 0.01	0.66 ± 0.03
SL	MIL + RNN	insp 0%	NHW + AA all	NHW matched	0.56 ± 0.04	0.53 ± 0.05
SL	MIL + RNN	insp 0%	NHW + AA all	AA	0.65 ± 0.03	0.62 ± 0.04
SL	PatchClass + RNN	insp 0%	NHW matched	NHW matched	0.53 ± 0.04	0.67 ± 0.01
SL	PatchClass + RNN	insp 0%	NHW matched	AA	0.53 ± 0.04	0.67 ± 0.01
SL	PatchClass + RNN	insp 0%	AA	NHW matched	0.61 ± 0.03	0.63 ± 0.01
SL	PatchClass + RNN	insp 0%	AA	AA	0.65 ± 0.03	0.67 ± 0.02

SL	PatchClass + RNN	insp 0%	NHW + AA balanced	NHW matched	0.63 ± 0.08	0.66 ± 0.03
SL	PatchClass + RNN	insp 0%	NHW + AA balanced	AA	0.62 ± 0.07	0.67 ± 0.29
SL	PatchClass + RNN	insp 0%	NHW + AA all	NHW matched	0.61 ± 0.03	0.58 ± 0.00
SL	PatchClass + RNN	insp 0%	NHW + AA all	AA	0.65 ± 0.03	0.64 ± 0.03
SSL	cOOpD (SimCLR)	insp 0%	NHW matched	NHW matched	0.80 ± 0.00	0.76 ± 0.00
SSL	cOOpD (SimCLR)	insp 0%	NHW matched	AA	0.76 ± 0.00	0.64 ± 0.00
SSL	cOOpD (SimCLR)	insp 0%	AA	NHW matched	0.81 ± 0.00	0.76 ± 0.01
SSL	cOOpD (SimCLR)	insp 0%	AA	AA	0.78 ± 0.00	0.70 ± 0.01
SSL	cOOpD (SimCLR)	insp 0%	NHW + AA balanced	NHW matched	0.82 ± 0.00	0.78 ± 0.00
SSL	cOOpD (SimCLR)	insp 0%	NHW + AA balanced	AA	0.80 ± 0.01	0.75 ± 0.01
SSL	cOOpD (SimCLR)	insp 0%	NHW + AA all	NHW matched	0.83 ± 0.00	0.80 ± 0.00
SSL	cOOpD (SimCLR)	insp 0%	NHW + AA all	AA	0.81 ± 0.00	0.74 ± 0.00
SSL	NNCLR	insp 0%	NHW matched	NHW matched	0.73 ± 0.00	0.69 ± 0.00
SSL	NNCLR	insp 0%	NHW matched	AA	0.71 ± 0.00	0.59 ± 0.00
SSL	NNCLR	insp 0%	AA	NHW matched	0.78 ± 0.00	0.75 ± 0.00
SSL	NNCLR	insp 0%	AA	AA	0.73 ± 0.00	0.64 ± 0.00
SSL	NNCLR	insp 0%	NHW + AA balanced	NHW matched	0.73 ± 0.00	0.69 ± 0.00
SSL	NNCLR	insp 0%	NHW + AA balanced	AA	0.70 ± 0.00	0.59 ± 0.01
SSL	NNCLR	insp 0%	NHW + AA all	NHW matched	0.73 ± 0.00	0.68 ± 0.00
SSL	NNCLR	insp 0%	NHW + AA all	AA	0.71 ± 0.00	0.59 ± 0.00
SSL	cNNCLR	insp 0%	NHW matched	NHW matched	0.78 ± 0.00	0.66 ± 0.00
SSL	cNNCLR	insp 0%	NHW matched	AA	0.72 ± 0.00	0.55 ± 0.00
SSL	cNNCLR	insp 0%	AA	NHW matched	0.78 ± 0.00	0.75 ± 0.00
SSL	cNNCLR	insp 0%	AA	AA	0.79 ± 0.00	0.70 ± 0.00
SSL	cNNCLR	insp 0%	NHW + AA balanced	NHW matched	0.81 ± 0.00	0.74 ± 0.00

SSL	cNNCLR	insp 0%	NHW + AA balanced	AA	0.81 ± 0.00	0.67 ± 0.00
SSL	cNNCLR	insp 0%	NHW + AA all	NHW matched	0.85 ± 0.00	0.76 ± 0.00
SSL	cNNCLR	insp 0%	NHW + AA all	AA	0.81 ± 0.00	0.69 ± 0.01

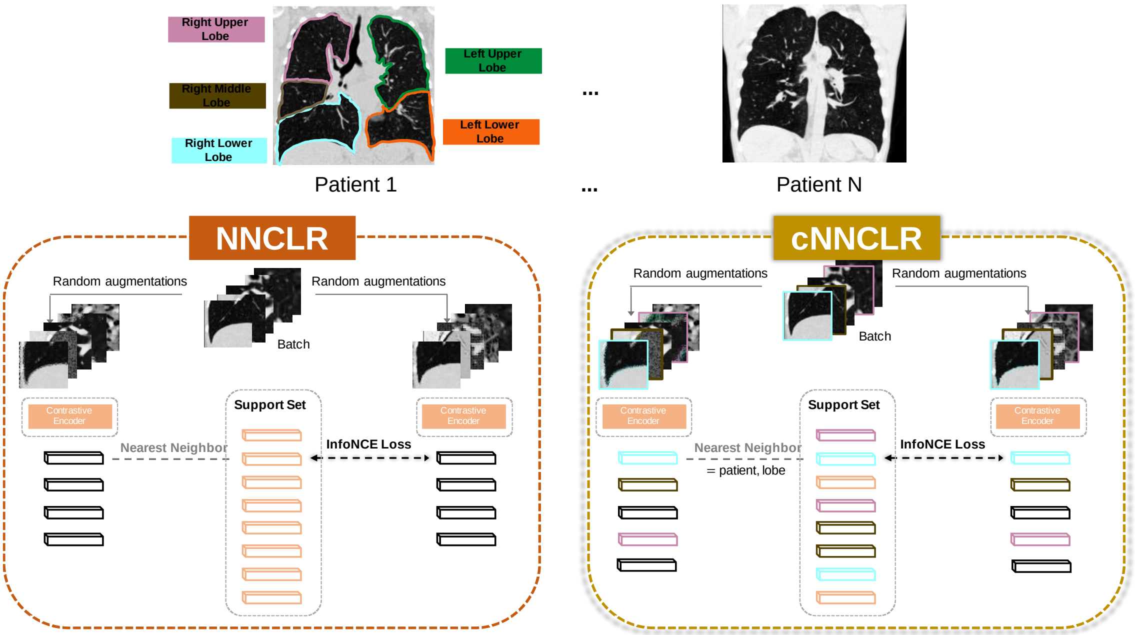

Supplementary figure S1: Differences between the Nearest-Neighbor Contrastive Learning (NNCLR) framework and the Context-Aware NNCLR (cNNCLR) adaptation.
In the Nearest-Neighbor Contrastive Learning (NNCLR) framework, each patch undergoes augmentation twice with random transformations. One view is replaced with its nearest neighbor (NN) in the latent space, sampled from a memory bank (support set) updated with every batch. This approach aims to introduce diversity in positive pairs by leveraging nearest neighbors, thereby enriching the latent representation beyond single instances. The Context-Aware NNCLR (cNNCLR) adaptation enhances NNCLR by enforcing the nearest neighbor to originate from the same lung lobe and the same patient within the memory bank. This ensures that nearest neighbors are selected based on spatial and disease-related considerations, improving the model's ability to capture subtle and heterogeneous pathological patterns specific to lung lobe regions.
